# Themes of Holism and Reductionism in the Quest for the Cause of Tuberculosis

**DOI:** 10.3201/eid3103.AC3103

**Published:** 2025-03

**Authors:** Terence Chorba

**Keywords:** tuberculosis, Hans Schadow, Rudolph Virchow, Robert Koch, Koch’s disease, Mycobacterium tuberculosis, tuberculosis and other mycobacteria, art and science, about the cover

**Figure:**
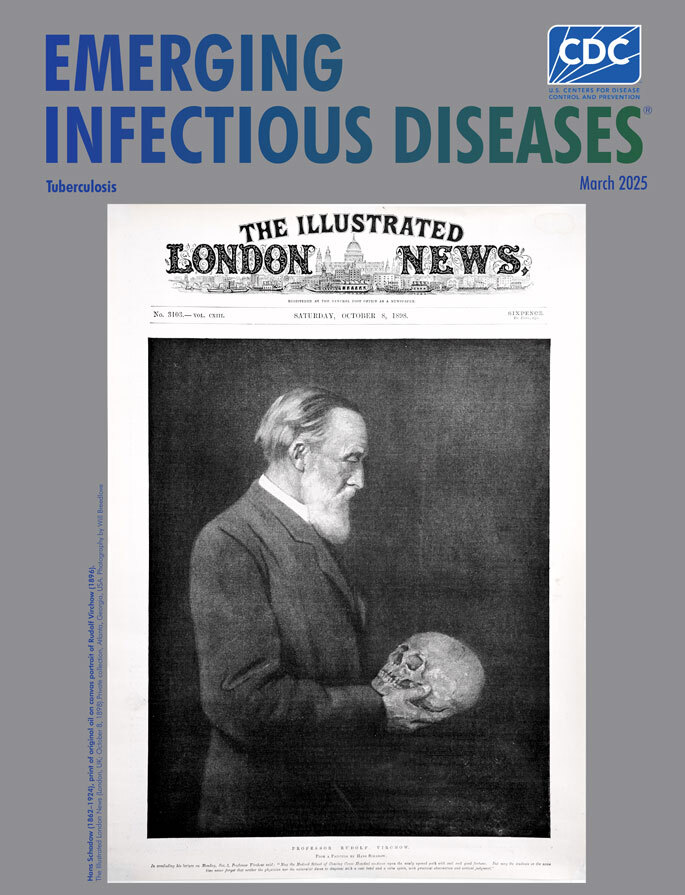
**Hans Schadow (1862–1924), print of original oil on canvas portrait of Rudolf Virchow (1896).** *The Illustrated London News* (London, UK; October 8, 1898). Private collection, Atlanta, Georgia, USA. Photography by Will Breedlove.

In 1882, at a time when many prominent physicians, including Rudolf Virchow (1821–1902), had yet to accept the germ theory of disease, 2 contrasting but complementary schools of thought on the etiology of tuberculosis (TB) were dominant. The first was “holistic,” focused principally on the host (susceptibility of the exposed individual) and the environment (extrinsic factors that affect the opportunity for exposure and development of disease); its leading proponent was Virchow, a German physician whose seminal work in cellular pathology, social medicine, and public health contributed substantially to the development of modern medicine. The second was “reductionistic,” focused on identifying the agent (the organism or event that resulted in disease or disability) and on deconstructing the complex process of infection into its component parts; its leading proponent was Robert Koch (1843–1910), whose crowning achievement—the discovery of the organism—is celebrated annually worldwide in March and is the impetus for dedicating each March issue of this journal to tuberculosis.

Virchow popularized the biological principle that every cell originates from a predecessor cell (omnis cellula e cellula in Latin, a theory originally proposed by physiologist Theodor Schwann and botanist Matthias Schleiden) and held that all disease involves changes in normal cells (i.e., all pathology is cellular pathology). Among Virchow’s many achievements as “the father of modern pathology” were the first descriptions of many medical phenomena including leukemia, embolism, and thrombosis. Virchow was also a strong proponent of social medicine, advancing insightful arguments that disease was often caused and exacerbated by social and economic conditions, rather than just biological factors. His belief in the social determinants of health led him to identify nutrition, poverty, and sanitation as influential factors in public health and to advocate for improvements in living and working conditions.

On the evening of March 24, 1882, at the Berlin Institute of Physiology, Robert Koch presented his conclusive work demonstrating that infection with a characteristic bacillus (*Mycobacterium tuberculosis*) was the cause of TB. Koch’s conclusion was based on strict criteria or postulates now considered essential for establishing scientific consensus that a given microorganism causes a disease: 1) the organism can be shown to be consistently present in diseased tissue; 2) the organism can be isolated and grown in pure culture; and 3) the organism can be shown experimentally to induce the disease in animal models. The Nobel Prize–winning physician and chemist Paul Ehrlich (1854–1915), who had been an early assistant of Koch and a TB survivor himself, later said of that evening’s meeting that it had been “the most important experience of my scientific life.”

Koch had much ground to cover in his brief presentation of that reductionist science (i.e., proving TB to be a specific disease with a specific infectious etiology). Although Virchow was in attendance, he remained silent. As a proponent of the societal and environmental causes of disease, Virchow had characterized TB as a social disease strongly linked to poverty and had disputed Koch’s germ theory. His holistic recognition of the social determinants of health constituted the basis for advocacy of comprehensive health reforms (i.e., improved housing, sanitation, water supply, and working conditions). In the years immediately following Koch’s discovery, there was great interest in seeking nonexclusionary remedies that addressed both the holistic and reductionist views for TB prevention and control. Holistic efforts attempted to avert the societal burden of transmission of the bacillus through public health campaigns to raise awareness of risk factors and to improve living and working conditions. Reductionist efforts were aimed at attenuating the bacillus through use of serum, mostly from infected animals and humans. Both Koch and Virchow achieved great public stature in their lifetimes for their contributions to medical science and public health.

In 1896, Hans Schadow (1862–1924), an accomplished portrait painter born in Berlin, Germany, created a detailed oil-on-canvas portrait of Rudolf Virchow contemplating a human skull; the portrait was commissioned by *The Illustrated London News*, a magazine published weekly during 1842–1971 and then less frequently before ceasing publication altogether in 2003. The magazine itself was revolutionary, as the first fully illustrated weekly newspaper, and took the occasion of Virchow’s delivering a medical lecture in London to reproduce Schadow’s painting as a print on its front page in 1898, featured again on the cover of this month’s journal.

In modern-day TB elimination efforts, effective prevention and control programs have several priority strategies that include identifying and completing treatment of TB disease; finding and screening persons who have been recent contacts of TB patients; and screening, testing, and treating populations at greater risk of having latent TB infection and of developing TB disease. Components of Koch’s reductionist approach are fundamental to the identification of persons with TB disease; although a minority of incident TB cases have no bacilli detectable in sputum or in other body fluids, TB disease is usually confirmed by a positive culture or nucleic acid amplification test result for *M. tuberculosis.* However, components of Virchow’s holistic approach are essential for identifying, testing, and treating populations at greater risk and for whom testing and treatment are most defensible in a resource-limited environment (e.g., persons from high-burden countries; those who lived or worked with persons with TB disease; or those with immune compromise resulting from diabetes, smoking, alcohol use disorder, cancer, or HIV infection). Both perspectives have intrinsic relevance to the strategies of health departments in TB elimination efforts.
